# Arrhythmogenic right ventricular cardiomyopathy (ARVC): cardiovascular magnetic resonance update

**DOI:** 10.1186/s12968-014-0050-8

**Published:** 2014-07-20

**Authors:** Anneline SJM te Riele, Harikrishna Tandri, David A Bluemke

**Affiliations:** 1 Department of Medicine, Division of Cardiology, University Medical Center Utrecht, Utrecht, The Netherlands; 2 Department of Medicine, Division of Cardiology, Johns Hopkins University School of Medicine, Baltimore, MD, USA; 3 Department of Radiology, Johns Hopkins University School of Medicine, Baltimore, MD, USA; 4 Radiology and Imaging Sciences, National Institutes of Health Clinical Center, 10 Center Drive, Bethesda 20892, MD, USA

**Keywords:** Arrhythmogenic right ventricular cardiomyopathy, Cardiovascular magnetic resonance, Diagnosis, Genetics, Treatment

## Abstract

Arrhythmogenic Right Ventricular Cardiomyopathy (ARVC) is one of the most arrhythmogenic forms of inherited cardiomyopathy and a frequent cause of sudden death in the young. Affected individuals typically present between the second and fourth decade of life with arrhythmias coming from the right ventricle. Pathogenic mutations in genes encoding the cardiac desmosome can be found in approximately 60% of index patients, leading to our current perception of ARVC as a desmosomal disease. Although ARVC is known to preferentially affect the right ventricle, early and/or predominant left ventricular involvement is increasingly recognized. Diagnosis is made by combining multiple sources of diagnostic information as prescribed by the “Task Force” criteria. Recent research suggests that electrical abnormalities precede structural changes in ARVC. Cardiovascular Magnetic Resonance (CMR) is an ideal technique in ARVC workup, as it provides comprehensive information on cardiac morphology, function, and tissue characterization in a single investigation. Prevention of sudden cardiac death using implantable cardioverter-defibrillators is the most important management consideration. This purpose of this paper is to provide an updated review of our understanding of the genetics, diagnosis, current state-of-the-art CMR acquisition and analysis, and management of patients with ARVC.

## Introduction

Arrhythmogenic right ventricular cardiomyopathy (ARVC) is an inherited cardiomyopathy characterized by fibro-fatty replacement of predominantly the right ventricular (RV) myocardium, which predisposes patients to life-threatening ventricular arrhythmias and RV dysfunction [1]–[3]. ARVC is present in up to 20% of individuals who experience sudden cardiac death (SCD) before the age of 35 years and is even more common among athletes who die suddenly [2],[4]. The disease has been reported to have prevalence of 1 in 2000 to 5000 individuals, although some reports estimate the real prevalence could be as high as 1 in 1000 in certain regions of the world due to under-recognition [5],[6]. Over the past decade, genetic testing for ARVC-associated mutations in five desmosomal genes and several non-desmosomal genes has become clinically available [7]. Inheritance is typically autosomal dominant with incomplete penetrance and variable expressivity [7]–[9]. Affected patients classically present between the second and fourth decade of life with ventricular arrhythmias coming from the RV [3]. However, SCD can occur as early as in adolescence, whereas mutation carriers may also remain asymptomatic throughout life [3],[10],[11].

Imaging modalities commonly used for ARVC evaluation include echocardiography, cardiovascular magnetic resonance (CMR), and RV angiography. Both echocardiography and angiography have significant limitations in assessing the RV due to its complex geometry [12]. Over the last decade, CMR has emerged as the imaging modality of choice in ARVC, allowing for non-invasive morphological and functional evaluation, as well as tissue characterization in a single investigation [13],[14]. In spite of its low prevalence, ARVC accounts for a disproportionately high percentage of referrals for CMR. Unfortunately, many imaging centers have little experience with evaluating ARVC, and gaining experience is difficult because of the low prevalence of disease. The aim of this review is to review current knowledge of ARVC that is useful for CMR interpretation in ARVC. Our emphasis will be on an update of issues relating to CMR diagnosis of ARVC [15], including ARVC diagnostic criteria and common regional morphological and functional abnormalities in this disease.

## Update on ARVC diagnosis

Diagnosis of ARVC may be challenging, as no single modality is sufficiently specific to establish ARVC diagnosis. Therefore, multiple sources of diagnostic information are combined in a complex set of diagnostic criteria. The original “Task Force” criteria (TFC), described in 1994 [16], largely relied on qualitative parameters and were shown to be insensitive to the disease especially in early stages [17]–[20]. In addition, imaging criteria were not specific, and led to many false positive diagnoses.

In 2010, modifications to the criteria were proposed (Table 1) [21]. These modifications had two purposes: (1) To improve the specificity of the diagnostic criteria by including quantitative metrics for ARVC diagnosis, and (2) To improve sensitivity of diagnosis in individuals who have a high likelihood of inherited/genetic disease. Specifically, quantitative parameters were included for imaging criteria, endomyocardial biopsy, and (signal-averaged) ECG. In addition, the revised TFC now include an ARVC-associated pathogenic mutation as a major criterion towards ARVC diagnosis. These changes to the TFC have resulted in increased sensitivity for inherited/genetic disease, while maintaining satisfactory specificity [22]–[24].

**Table 1:** Revised 2010 Task Force Criteria for ARVC*

**1. Global or regional dysfunction and structural alterations**			
Major			
	2D Echo Criteria		
		Regional RV akinesia, dyskinesia, or aneurysm AND 1 of the following measured at end diastole:	
			- PLAX RVOT ≥ 32 mm (PLAX/BSA ≥ 19 mm/m^2^), or
			- PSAX RVOT ≥ 36 mm (PSAX/BSA ≥ 21 mm/m^2^), or
			- Fractional area change ≤ 33%
	CMR criteria		
		Regional RV akinesia or dyskinesia or dyssynchronous RV contraction AND 1 of the following:	
			- RV EDV/BSA ≥ 110 mL/m^2^ (male) or ≥ 100 mL/m^2^ (female)
			- RV ejection fraction ≤ 40%
	RV angiography criteria		
		Regional RV akinesia, dyskinesia, or aneurysm	
Minor			
	2D Echo Criteria		
		Regional RV akinesia or dyskinesia or dyssynchronous RV contraction AND 1 of the following measured at end diastole:	
			- PLAX RVOT ≥29 to <32 mm (PLAX/BSA ≥16 to <19 mm/m^2^), or
			- PSAX RVOT ≥32 to <36 mm (PSAX/BSA ≥18 to <21 mm/m^2^), or
			- Fractional area change > 33% ≤ 40%
	CMR criteria		
		Regional RV akinesia or dyskinesia or dyssynchronous RV contraction AND 1 of the following:	
			- RV EDV/BSA ≥100 to 110 mL/m^2^ (male) or ≥90 to 100 mL/m^2^ (female)
			- RV ejection fraction >40 to ≤ 45%
**2.Tissue characterization of wall**			
Major			
	Residual myocytes < 60% by morphometric analysis (or < 50% if estimated), with fibrous replacement of the RV free wall myocardium in ≥1 sample, with or without fatty replacement of tissue on endomyocardial biopsy		
Minor			
	Residual myocytes 60% to 75% by morphometric analysis (or 50% to 65% if estimated), with fibrous replacement of the RV free wall myocardium in ≥1 sample with or without fatty replacement of tissue on endomyocardial biopsy		
**3. Repolarization abnormalities**			
Major			
	Inverted T waves in right precordial leads (V1, V2, and V3) or beyond in individuals >14 yrs of age (in the absence of complete RBBB QRS ≥ 120 ms)		
Minor			
	Inverted T waves in V1 and V2 in individuals >14 yrs of age (in the absence of complete RBBB) or in V4, V5, and V6		
	Inverted T waves in leads V1, V2, V3, and V4 in individuals >14 years of age in the presence of a complete RBBB		
**4. Depolarization/conduction abnormalities**			
Major			
	Epsilon wave (reproducible low-amplitude signals between end of QRS complex to onset of T wave) in the right precordial leads (V1 - V3)		
Minor			
	Late potentials by SAECG in ≥1 of 3 parameters in the absence of a QRSd of ≥110 msec on standard ECG:		
	- Filtered QRS duration (fQRS) ≥ 114 msec		
	- Duration of terminal QRS < 40 microV ≥ 38 ms		
	- Root-mean-square voltage of terminal 40 ms ≤20 micro V		
	Terminal activation duration ≥ 55 ms measured from the nadir of the S-wave until the end of all depolarization deflections (including R') in V1, V2, or V3		
**5. Arrhythmias**			
Major			
	Nonsustained or sustained VT of LBBB morphology with superior axis		
Minor			
	Nonsustained or sustained VT of RVOT configuration, LBBB morphology with inferior axis or of unknown axis		
	> 500 PVCs per 24 hours on Holter monitoring		
**6. Family History**			
Major			
	ARVC in first degree relative who meets Task Force Criteria		
	ARVC confirmed pathologically at autopsy or surgery in first degree relative		
	Identification of pathogenic mutation categorized as associated or probably associated with ARVC in the patient under evaluation		
Minor			
	History of ARVC in first degree relative in whom it is not possible to determine whether the family member meets Task Force Criteria		
	Premature sudden death (<35 years of age) due to suspected ARVC in a first degree relative		
	ARVC confirmed pathologically or by current Task Force Criteria in second-degree relative		

## Role of CMR in ARVC diagnosis

### CMR protocol for ARVC

The CMR protocol that we recommend for ARVC evaluation shown in Table 2. The protocol has been designed to evaluate the RV for abnormalities in structure and tissue characterization while enabling quantitative evaluation. For black blood imaging, fast spin echo or turbo spin echo imaging sequences are ideal. The RV free wall and RV outflow tract are best evaluated in the axial black blood images. The stack of axial images should include the entire RV. This can be accomplished in 6–8 slices at intervals (slice thickness + gap) of about 1 cm. Most of the diagnostic information is obtained in the slices centered on the middle of the RV. Obtaining an excessive number of image slices will adversely prolong the examination.

**Table 2:** Recommended CMR protocol for ARVC

**Sequence**	**Imaging plane**	**Parameters**	**Comments**
*Double inversion recovery TSE/FSE a) Axial: with and without fat suppression b) short axis: without fat suppression*	a) Axial: obtain ~6-8 images centered on the left/right ventricle	TR = 2 R-R intervals, TE = 5 msec (minimum-full) (GE), TE = 30 msec (Siemens) slice thickness = 5 mm, interslice gap = 5 mm, and field of view (FOV) = 28–34 cm. ETL 16-24	This sequence provides optimal tissue characterization of the RV free wall. Prescribe from the pulmonary artery to the diaphragm. Fat suppression improves reader confidence in diagnosis of RV fat infiltration.
	b) Short axis: obtain ~6-8 images centered on the left ventricle		
*SSFP Bright Blood Cine Images*	Axial, Four chamber and Short Axis. RV 3 chamber (optional)	TR/TE minimum, flip angle = 45-70°, slice thickness = 8 mm, interslice gap = 2 mm. FOV = 36–40 cm, 16–20 views per segment. Parallel imaging n = 2 is desirable	Axial images are best to assess RV wall motion. RV quantitative analysis is performed on the short axis cine images.
**GADOLINIUM IS ADMINISTERED ACCORDING TO INSTITUTIONAL PROTOCOL (usually 0.15 – 0.2 MMOL/KG)**			
*TI scout*	Four chamber		TI scout sequences or trial TI times to suppress normal myocardium for the right inversion time.
*Delayed Gadolinium Imaging (Phase Sensitive Inversion Recovery recommended)*	Axial, Short Axis, Four Chamber and Vertical Long Axis	TR/TE per manufacturer recommendations flip angle = 20-25°, slice thickness = 8 mm, interslice gap = 2 mm. FOV = 36–40 cm, No parallel imaging. Use phase sensitive inversion recovery if available (PSIR)	PSIR is more robust and independent of TI time. Optimal for imaging fibrosis. LV epicardial enhancement in the infero-lateral wall has been reported in classic ARVC and in left dominant forms.

*Abbreviations*: *ARVC* Arrhythmogenic Right Ventricular Cardiomyopathy, *LV* left ventricle, *FOV* field of view, *FSE* fast spin echo, *PSIR* phase sensitive inversion recovery, *RV* right ventricle, *SSFP* steady state free precession, *TE* echo time, *TI* inversion time, *TR* repetition time, *TSE* Turbo spin echo.

For cine imaging, steady state free precession (SSFP) imaging is preferred at 1.5 Tesla. Insufficient information at 3 Tesla is available to determine if SSFP or fast gradient echo (FGRE) is superior. Quantitative analysis of the RV and left ventricle (LV) is performed on short axis images. Thus, 10–12 slices encompassing the entire ventricular volume must be obtained. We prescribe these images beginning approximately 1 cm above the valve plane and increment towards the apex of the ventricles. Cine images should also be obtained in standard long axis views of the LV. Some sites prefer to also acquire a vertical long axis view of the RV. Finally, we routinely obtain a stack of transaxial images of the RV at the same slice positions as the black blood images described above. Given modern CMR scanners, the temporal resolution of cine images is typically about 40 msec.

Delayed gadolinium images are best obtained using phase selective inversion recovery (PSIR), a sequence which does not depend upon identifying the precise inversion time (TI) [25]. In patients with significant ventricular ectopy, a low dose of a beta-blocker (metoprolol 25–50 mg) is recommended for arrhythmia suppression during the CMR scan.

### CMR TFC and their derivation

A major addition to the revised TFC was the inclusion of quantitative measurements for imaging criteria. The revised CMR TFC now require presence of both qualitative findings (RV regional akinesia, dyskinesia, dyssynchronous contraction) and quantitative metrics (decreased ejection fraction *or* increased indexed RV end-diastolic volume) (Table 1).

Quantitative values for RV volume and function for TFC were derived from a comparison of ARVC probands with normal healthy volunteers that were included in the Multi-Ethnic Study of Atherosclerosis (MESA) [26]. To ascertain cutoff values, RV dimension and function from 462 normal MESA participants were compared to 44 probands in the North American ARVC registry [21]. Major criteria (RV ejection fraction ≤40% *or* indexed RV end-diastolic volume ≥110 mL/m^2^ for men and ≥100 mL/m^2^ for women) were chosen to achieve approximately 95% specificity. Cutoffs with high specificity invariably result in lower sensitivity; major CMR criteria have a sensitivity of 68 to 76% [27]. Minor criteria (RV ejection fraction 40-45% *or* indexed RV end-diastolic volume 100–110 mL/m^2^ for men and 90–100 mL/m^2^ for women) had a higher sensitivity (79 to 89%), but a consequently lower specificity (85 to 97%) [27].

### Impact of new TFC on diagnostic yield

Several studies report on the impact of the revised TFC on diagnostic yield specifically for CMR [22],[28]–[30]. Unanimously, these studies showed a decrease in the prevalence of major and minor CMR criteria in the modified TFC compared to the original TFC. This corresponded to a decrease in sensitivity in most of these studies [22],[29]. Interestingly, although sensitivity decreased, the positive predictive value (PPV) increased with the revised TFC, as shown by Femia *et al.* (PPV increase from 23% in original criteria to 55% in revised criteria) [30]. This may largely be due to the inclusion of quantitative CMR criteria. Vermes *et al.* report that 97% of subjects with minor changes according to the original CMR criteria did not meet revised CMR criteria [29]. In addition, a low sensitivity of CMR for ARVC diagnosis is understandable in the context of the recently published data indicating that electrical abnormalities precede structural changes detected by CMR in ARVC [31]–[33]. This emphasizes the concept that ARVC evaluation should not be solely based on any one test, in particular CMR.

### Limitations of quantitative evaluation of the right ventricle: the revised task force criteria

Including quantitative metrics as a component to the CMR TFC has been an important contribution to ARVC evaluation, however some limitations exist. First, although quantitative measures reduce subjectivity of CMR TFC, there is substantial inter-reader variability for the RV. In our experience, two physician readers had excellent agreement (within ~5% of reference values) only after training on approximately 100 CMR cases [34]. In clinical practice, we expect it would be difficult to achieve reproducibility of less than 10% for RV parameters.

Cutoffs for ARVC criteria were derived from the MESA study that used the FGRE technique whereas a majority of study subjects in the US ARVC study had SSFP cine images. RV volumes by SSFP are least 10% larger than those measured with FGRE technique [35]–[39]. SSFP images provide superior contrast between blood and endocardium at the endocardial border, with less blood flow dependence [35],[36],[40]. Also, the revised TFC used MESA subjects whose mean age was 60 years. ARVC subjects in the Task Force study were 20–30 years younger on average. Since that time, Chahal *et al.* determined that, among MESA participants aged 45 to 84 years, RV end-diastolic volume decreased 4.6% per decade [34]. A very similar percentage of approximately 4% decrease per decade was obtained by Maceira *et al.* using the SSFP technique in subjects 20 to 80 years old [41]. Adjusting for body surface area did not remove the dependence of RV volume on age.

Fortuitously, the issues pulse sequence and older reference population in the MESA approximately balance each other. As an example, the CMR cutoff values for RV size in ARVC (Table 1) are ≥110 mL/m2 (male) or ≥100 mL/m2 (female) for major criteria. These values closely correspond to the 95^th^ percentile confidence limits of RV volumes for normal subjects less than 60 years old [41]. Thus, until further studies are available, we feel that the current RV metrics in the revised TFC remain highly relevant. As important, further developments are necessary to improve the reproducibility of RV quantification by CMR. When evaluating younger patients, CMR physicians should keep in mind that the size of the RV is expected to be ~10% larger in a 20 year old compared to a 40 year old patient.

## Common findings in ARVC by CMR

**Figure 1:**
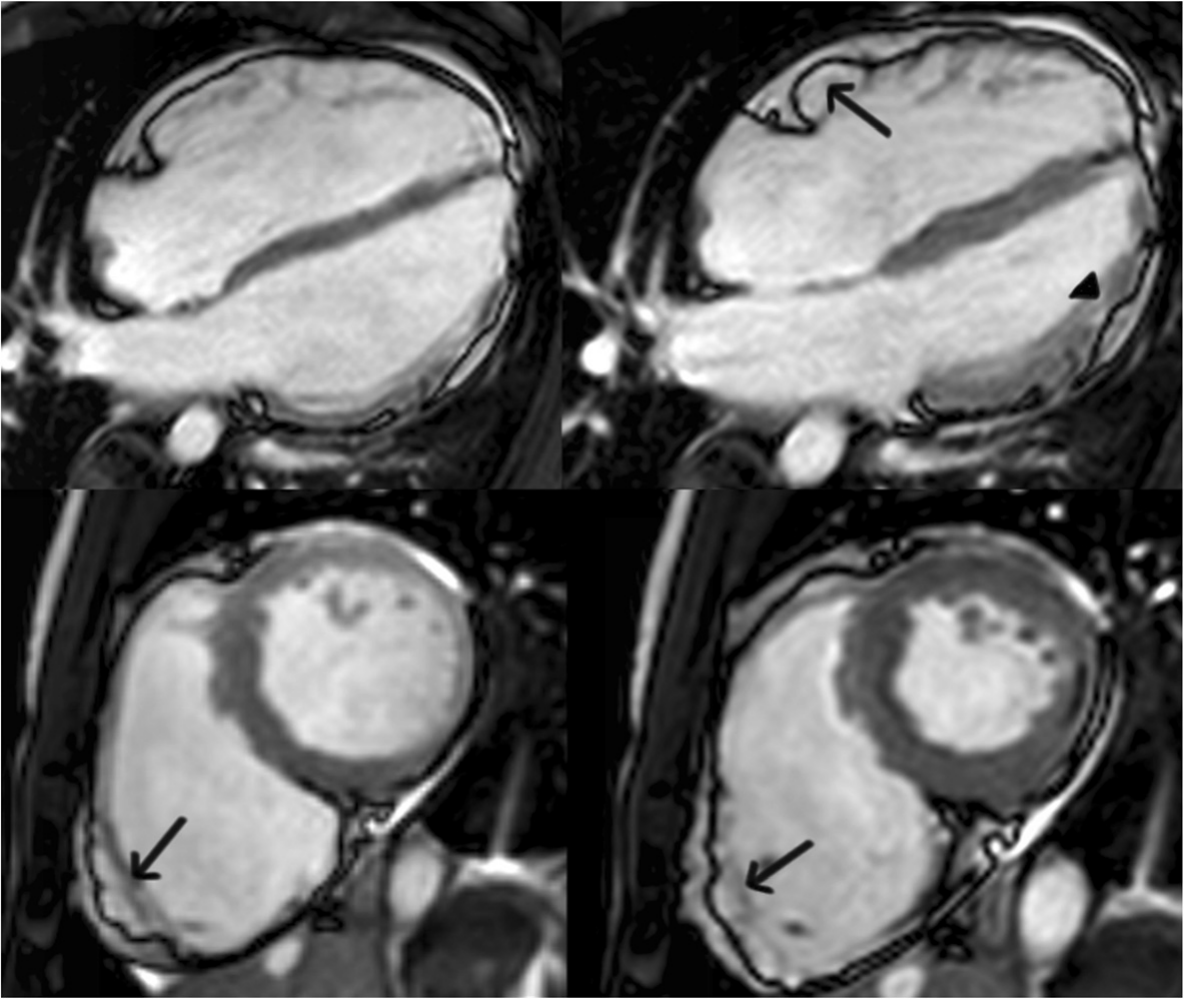
**Four-chamber (top panels) and short-axis (bottom panels) bright blood images in an ARVC subject with predominant right ventricular abnormalities.** End-diastolic images are shown in the left panels, end-systolic images in the right panels. Note subtricuspid dyskinesia in the end-systolic four-chamber image (arrow), and right ventricular free wall aneurysms (i.e. both systolic and diastolic bulging) in the short-axis image (arrows).

Most of our information about structural abnormalities in ARVC comes from studies in subjects with a predominant RV phenotype (Figure 1) [42]–[44]. Abnormalities in the RV in ARVC have been extensively described (reviewed in [15]). Besides global reduction in RV function and enlargement of the RV, more subtle regional disease of the RV has been variously described in the literature using a variety of terms (including focal bulges, microaneurysms, segmental dilatation, regional hypokinesis, etc.). In the current TFC, the terms “akinesia” (lack of motion) and “dyskinesia” (abnormal movement – instead of contracting in systole, that segment of myocardium bulges outward in systole) and “dyssynchronous” (regional peak contraction occurring at different times in adjacent myocardium) are used for all imaging modalities (CMR, echocardiography and angiography) to describe regional wall motion abnormalities in ARVC. Microaneurysms are not explicitly described in the revised TFC for CMR; overuse of this finding was considered by the Task Force members to be misinterpreted by CMR physicians resulting in false positive diagnoses. However, microaneurysms are characterized by regional akinesia or dyskinesia in the revised criteria.

**Figure 2:**
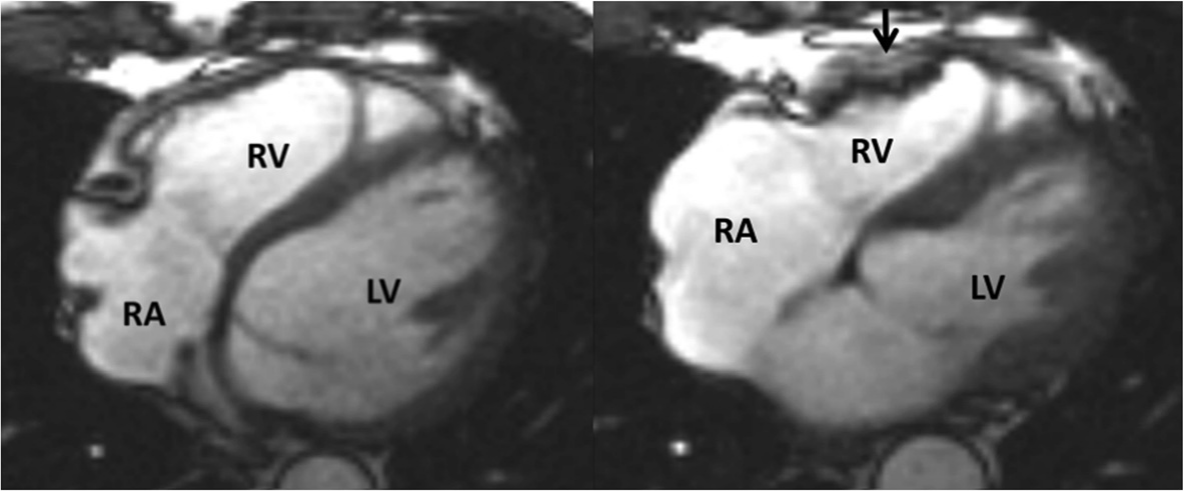
**Regional contraction abnormality in the subtricuspid region.** End diastolic (left) and end systolic image (right) show the so-called “accordion sign” in an ARVC mutation carrier. Regional dyssynchronous contraction in the subtricuspid region is a readily recognized qualitative pattern of abnormal RV contraction.

The location of regional wall motion abnormalities of the RV was not addressed in the revised TFC. We now recognize that the distal RV (from the moderator band to the apex in long axis views) shows highly variable contraction patterns in the normal individual. Therefore in ARVC, we emphasize the significance of regional wall motion abnormalities in the subtricuspid region. An excellent example of this is the so-called “accordion sign” that represents a focal “crinkling” of the myocardium (Figure 2) [45],[46]. In terms of TFC, the accordion sign is due to a small region of highly localized myocardium with dyssynchronous contraction.

### The changing spectrum of ARVC: The triangle of dysplasia displaced

Since the first report in 1982, regional abnormalities in ARVC were thought to locate to the RV inflow tract, outflow tract, and apex, collectively referred to as the “Triangle of Dysplasia” [1]. This concept was based on RV angiographic findings and autopsy data in a series of 24 patients with ARVC. In subsequent years, studies describing structural changes in ARVC focused on abnormalities in these three regions [47]. It is important to note that these observations were made in tertiary centers, without the advantage of genetic testing, and without sensitive TFC.

The last decade has witnessed a paradigm shift in our perception of regional structural involvement in ARVC. Autopsy series have shown predominant fibro-fatty infiltration on the epicardial surface, suggesting that the disease starts in the epicardium and progresses to the endocardium [47]. In 2004, Marchlinski *et al.* were one of the first to note preferential subtricuspid involvement in ARVC [48]. These results were confirmed in multiple studies using CMR [49],[50], echocardiography [51], and electroanatomic voltage mapping [50],[52],[53].

Recently, Te Riele *et al.* provided a series of 80 ARVC mutation carriers who underwent CMR and/or endo- and epicardial electroanatomic voltage mapping [54]. Structural abnormalities in this cohort preferentially located to the epicardial subtricuspid region and basal RV free wall, whereas the RV apex and endocardium were relatively spared. In addition, the authors reported that the LV lateral wall was significantly more often involved than the RV apex, especially among subjects with early disease. This led the authors to coin the “displacement” of the RV apex from the Triangle of Dysplasia [54]. Although preferential involvement of the subtricuspid region and LV lateral wall has been described before in separate ARVC reports, the focus on sparing of the RV apex is novel. This is particularly important, as the RV apex is often thin and difficult to assess, resulting in a frequent cause of misdiagnosis in ARVC.

### The changing spectrum of ARVC: Left ventricular involvement

**Figure 3:**
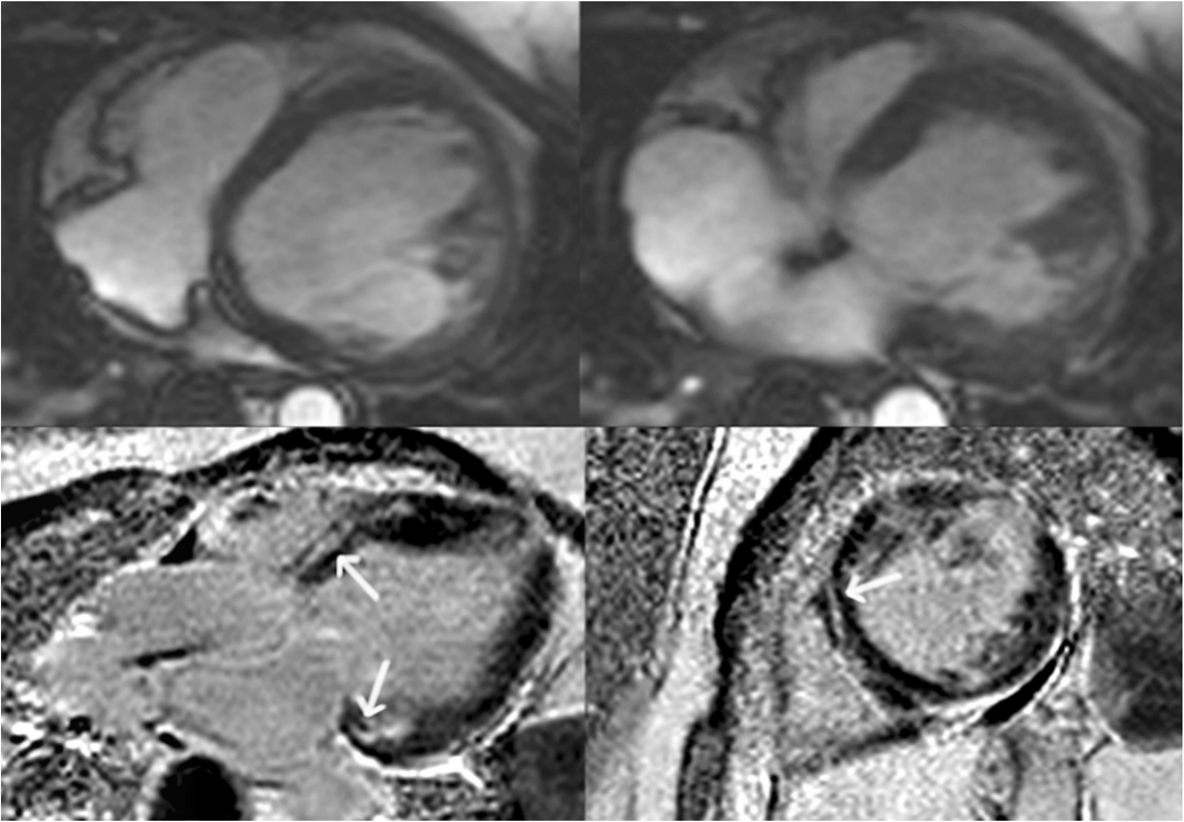
**Horizontal long-axis (top panels) bright blood and late gadolinium enhancement images (bottom panels) in an ARVC subject with predominant left ventricular abnormalities.** Note a dilated left ventricle in the bright blood images. Late enhancement is observed in a mid-myocardial pattern in the basal septum and basal lateral wall (arrows, bottom panels).

The advent of genetic testing and use of sensitive TFC have significantly enhanced our appreciation of the wide phenotypic spectrum of ARVC, and increased our awareness of non-classical (including left-dominant and biventricular) phenotypes. As a result, we now know that some ARVC subjects have early and predominant LV involvement (Figure 3) [19],[55]–[57]. LV involvement has even been reported in 76% of ARVC subjects, of whom the majority had advanced disease [58]. The disease is, therefore, increasingly being referred to as “Arrhythmogenic Cardiomyopathy”.

In 2010, Jain led a study investigating LV regional dysfunction using CMR tagging, and found that LV peak systolic strain was lower in ARVC subjects compared to controls [59]. Sen-Chowdhry *et al.* recently published data supporting a genetic association between left-dominant ARVC and classical right-sided ARVC [57]. In their study, the authors showed that one-third of genotyped ARVC patients with a left-dominant phenotype have a pathogenic mutation in the ARVC-related desmosomal genes. Phenotypic variations of predominant RV and LV involvement even coexisted in the same family.

**Figure 4:**
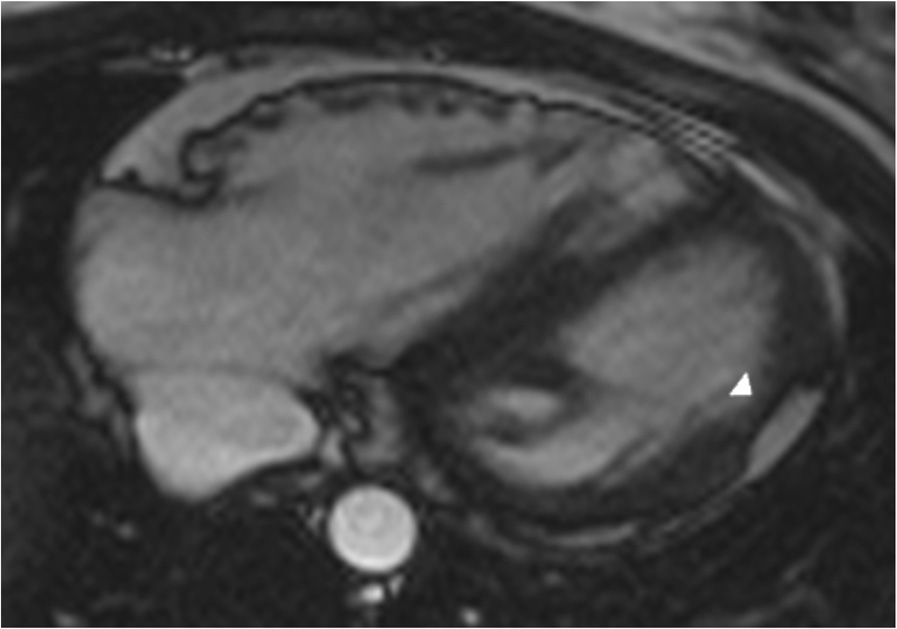
Horizontal long-axis bright blood image in an ARVC patient revealing left ventricular lateral wall fatty infiltration with myocardial wall thinning (arrowhead).

LV involvement in ARVC may manifest as late gadolinium enhancement (LGE), often involving the inferior and lateral walls without concomitant wall motion abnormalities [55],[57],[60]. Septal LGE is present in more than 50% of cases with left dominant ARVC, in contrast to the right dominant pattern in which septal involvement is unusual [55]. In addition, LV fatty infiltration was shown to be a prevalent finding in ARVC, often involving the subepicardial lateral LV and resulting in myocardial wall thinning (Figure 4) [54],[61]. Early data by Dalal *et al.* already showed that LV fat in the lateral wall is very specific for ARVC mutation carriers [45]. Future studies are necessary to confirm these data, and further our understanding of LV abnormalities in ARVC.

### Late gadolinium enhancement in ARVC evaluation

Myocardial LGE is a well-validated technique for assessment of myocardial fibrosis. Given that one of the pathologic hallmarks of ARVC is fibro-adipose replacement of the myocardium [47], it is important to note that LGE is not incorporated in the current diagnostic TFC. Although the Task Force did recognize the presence of LGE in many patients with ARVC, several limitations withheld its inclusion in the diagnostic criteria. First, detection of LGE in the RV is greatly hampered by the thin RV wall. A high variability between centers resulted in limitations of LGE in the multi-center US ARVC study. In ARVC, RV wall thinning is pronounced [42], which makes the LGE technique less reliable than for the LV. Second, distinguishing fat from fibrosis by LGE sequences is challenging, which makes its interpretation highly subject to the CMR physician’s experience. Last, LV LGE is non-specific, and has a wide differential diagnosis. LGE may be observed in many mimics of ARVC, such as sarcoidosis [62], myocarditis [63], amyloidosis [64], and dilated cardiomyopathy (DCM) [65].

**Figure 5:**
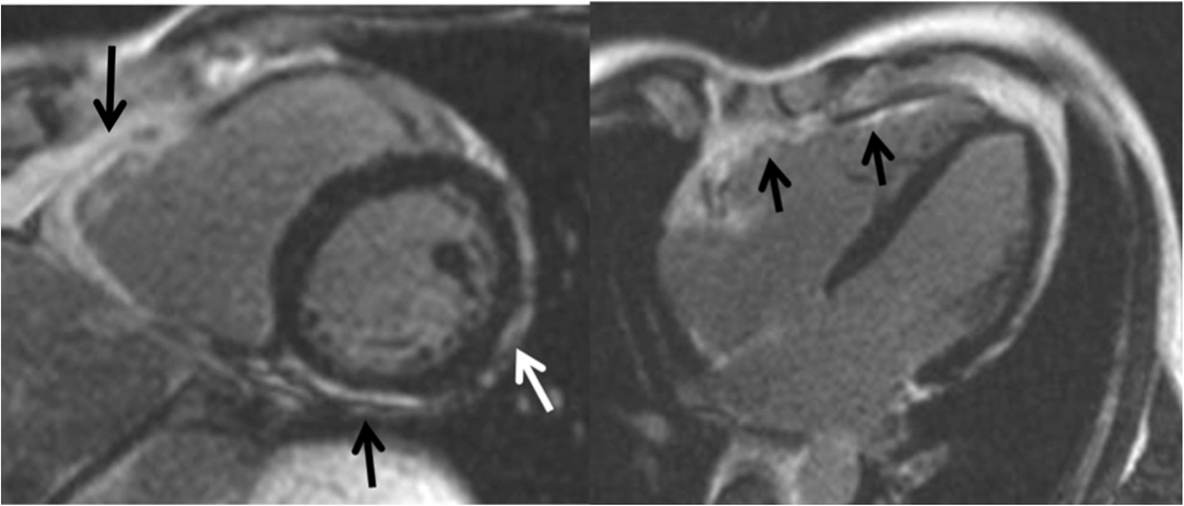
**Right ventricular late gadolinium enhancement in ARVC.** The short axis image (left) shows LGE in the RV as well as the LV (black arrows). The lateral wall of the LV shows thinning due to fatty replacement that was confirmed on T1-weighted images. The long axis view (right) shows diffuse LGE involving the free wall of the RV.

While these limitations exist, LGE may be very useful in ARVC evaluation (Figure 5). RV LGE has been observed in up to 88% of patients [66]–[68], while LV LGE was reported in up to 61% of cases [50],[69]. Importantly, before LGE can be included in a future iteration of the TFC, more data regarding the specific patterns of LGE that distinguish ARVC from other cardiomyopathies is necessary. Also, improved methods to determine fibrosis in the thin RV wall are needed. Until such a method emerges, use of LGE in clinical practice should be considered as diagnostic confirmation, not sole evidence of ARVC disease expression. LGE CMR is also extremely useful when ARVC is excluded due to other cardiomyopathy such as sarcoidosis.

LGE may also be useful in management of ARVC patients. Tandri *et al.* showed excellent correlation of RV LGE with histopathology and inducible ventricular arrhythmias on electrophysiologic study [68]. As such, identification of LGE by CMR may provide guidance for electrophysiologic studies and endomyocardial biopsy. However, it is important to note that recent studies correlating LGE with electroanatomic scar revealed that LGE is less sensitive for the detection of low voltage areas than endocardial mapping during electrophysiologic study [50],[70].

### Misdiagnosis of ARVC using CMR

Misdiagnosis of ARVC is a well-recognized problem. A prior study has shown that more than 70% of patients who were referred to Johns Hopkins Hospital from outside institutions with a diagnosis of ARVC did not actually meet diagnostic TFC [71]. In many cases, CMR misinterpretation is the cause of over-diagnosis in ARVC [71],[72]. It is important to realize that, although CMR may be regarded the standard of reference for evaluation of RV morphology and function, the use of CMR alone is not the “gold standard” for ARVC diagnosis. Rather, the TFC prescribe the use of multiple diagnostic tests. Great caution must be employed when the only abnormality in a presumed ARVC patient is found on CMR, as it is uncommon for ARVC patients to have a normal ECG and Holter monitor but an abnormal CMR [32].

A proper understanding of common CMR abnormalities and patterns of disease in ARVC is invaluable for accurate CMR evaluation. Previous reports have extensively focused on fibro-fatty myocardial replacement, wall thinning, RVOT enlargement, and RV dilatation and dysfunction in ARVC [42]–[44]. As one of the pathologic hallmarks of ARVC, intramyocardial fat accumulation was thought to be highly sensitive for the disease. Unfortunately, multiple reports have shown that intramyocardial fat was not reproducible even among experienced readers, constituting an important cause of misdiagnosis in ARVC [44],[71],[73],[74].

**Figure 6:**
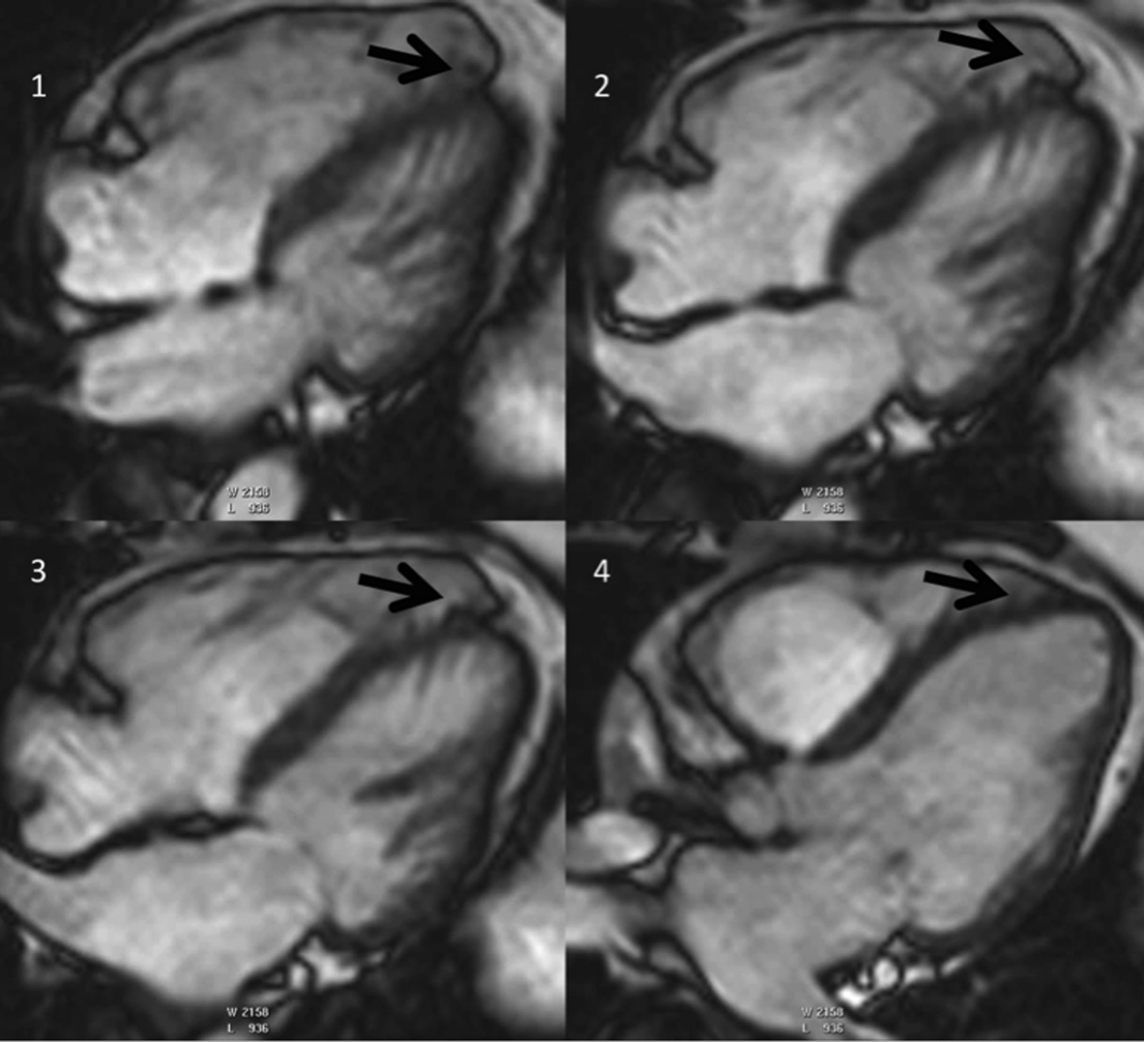
**Butterfly apex as a normal variation.** Stack of horizontal long axis views from inferior (image 1) to superior (image 4) in a control subject. Note the appearance of a butterfly apex on inferior views (arrows in images 1–3). This appearance is not seen on the more superior view (panel 4).

**Figure 7:**
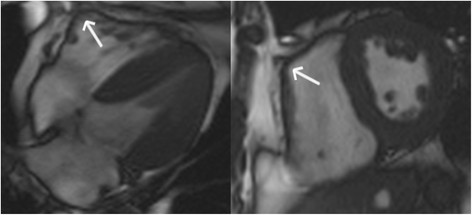
**Misdiagnosis of ARVC - Axial and short-axis bright blood images in a control subject.** Note the “tethering” of the mid right ventricular free wall to the anterior chest wall (arrows), giving the right ventricle a dyskinetic appearance.

Furthermore, normal variants as well as other pathologic conditions may mimic ARVC. Important normal variants that were previously mistaken for ARVC are pectus excavatum [75], apical-lateral bulging of the RV free wall at the insertion of the moderator band [76], and the “butterfly apex”, a normal anatomical variant of separate RV and LV apices causing the RV apex to look dyskinetic [77]. We have found the butterfly appearance of the apex to be more common on horizontal long axis views at inferior levels (Figure 6). In addition, a prominent band of pericardial connective tissue that joins the RV free wall to the posterior sternum may lead to misinterpretation of RV wall motion: this “tethered” portion of the RV free wall remains static in location and may be misinterpreted as RV dyskinesia (Figure 7). Additionally, pathologic disorders such as myocarditis and sarcoidosis may mimic ARVC [62],[63],[78]. Further testing to specifically exclude these conditions should be strongly considered, especially in the presence of LV dysfunction [79].

## Update on ARVC genetics

### ARVC: a desmosomal disease

Beginning with the seminal discovery of plakoglobin in 2000 [80], the past decade has witnessed the identification of mutations in five genes encoding the cardiac desmosome [81]–[84]. In recent reports, desmosomal mutations are found in up to 60% of ARVC cases [8],[23],[24],[85]. Among US ARVC patients, the most common gene involved is plakophilin-2 [85], followed by desmoglein-2, desmocollin-2, and desmoplakin [86]. Prevalence of mutations is similar in Europe [8],[87], although desmoplakin mutations are more prevalent in the United Kingdom and Italy [10],[57]. Desmosomes are complex multiprotein structures providing mechanical [88] and electrical [89] continuity to adjacent cells. Mechanical uncoupling in ARVC is accompanied by cell death and regional fibrosis, which causes the monomorphic arrhythmias typically associated with ARVC. In addition, electrical uncoupling through gap junction remodeling and sodium channel dysfunction may lead to significant activation delay [90],[91], which increases the propensity to functional block and arrhythmia. The exact mechanism by which these mutations cause the highly arrhythmogenic phenotype in ARVC has been subject of many hypotheses, which are extensively reviewed elsewhere [20],[47]. The List of Genes Section shows an overview of genes associated with ARVC.

### Genes associated with arrhythmogenic right ventricular dysplasia/cardiomyopathy

Gene

Desmosomal

 Desmocollin-2 (*DSC2*)

 Desmoglein-2 (*DSG2*)

 Desmoplakin (*DSP*)

 Junctional Plakoglobin (*JUP*)

 Plakophilin-2 (*PKP2*)

Non-Desmosomal

 Desmin (*DES*)

 Lamin A/C (*LMNA*)

 Phospholamban (*PLN*)

 Ryanodine Receptor (*RYR2*)

 Transmembrane Protein 43 (*TMEM43*)

 Titin (*TTN*)

### ARVC: overlap with other cardiomyopathies

In addition to desmosomal mutations, mutations in non-desmosomal genes have been identified in ARVC [92]–[98]. These non-desmosomal genes include, among others, desmin, titin, lamin A/C, and phospholamban, which are commonly mutated in subjects with DCM [99]–[101]. Although the distinction between ARVC and DCM has important implications for clinical practice, guiding both diagnostics and treatment, a considerable overlap of these conditions is increasingly recognized [102]. Compared to DCM, patients with left-dominant ARVC often have significant ventricular arrhythmias, disproportionate to the morphological abnormalities and impaired LV systolic function [57]. In addition, inflammatory processes such as (viral) myocarditis may mimic left-dominant ARVC [103]. In myocarditis, T2-weighted imaging may detect tissue edema, which is usually absent in ARVC [104]. In addition, fast spin-echo T1-weighted images during the first minutes after contrast injection may be useful to detect myocardial hyperemia and muscular inflammation suggestive of myocarditis [104],[105]. In equivocal cases, invasive studies such as electroanatomic mapping and endomyocardial biopsy may provide a more definite diagnosis [63].

### Impact of genetics on clinical ARVC management

With the identification of ARVC-causing mutations, integration of genetic testing into clinical practice is now proliferating. Currently, its main applications are confirmatory testing in index patients and cascade screening of families [106]. ARVC is generally transmitted as an autosomal dominant trait with incomplete penetrance and variable expressivity. A recent study by Cox *et al.* confirmed that asymptomatic mutation-carrying relatives have a 6-fold increased risk of developing ARVC compared to relatives of a proband without a pathogenic mutation [8]. However, it is important to realize that 50-70% of mutation carriers will never develop disease expression [7],[23],[107], and that severity of disease may vary greatly, even among members of the same family [11] or those carrying the same mutation [9]. In contrast, a negative genetic test result in a proband does not exclude the possibility of disease, nor does it exclude the possibility of a genetic process in the individual or family [106],[108]. Because of the complexities associated with interpreting genetic test results in ARVC, including genetic counseling prior and subsequent to genetic testing has been strongly recommended [106],[109].

### CMR in ARVC genotype-phenotype correlations

Over the last decade, several genotype-phenotype correlations in ARVC have been proposed, but large-scale studies confirming these observations are yet to come. Recently, patients with multiple desmosomal mutations were shown to have a more severe clinical course with more ventricular arrhythmias and more heart failure than subjects with a single mutation [10],[86]. In addition, individuals with a mutation in the phospholamban or desmoplakin gene (especially when involving the C-terminus of desmoplakin) have significant left-dominant/biventricular disease expression and a high prevalence of heart failure [19],[98],[110]. LV involvement in these patients often manifests as LGE in a LV circumferential, mid-myocardial pattern extending to the right side of the septum [111]. An example is shown in Figure 3. This left-dominant ARVC pattern should not be confused with LV involvement that occurs in advanced stages of right-dominant ARVC. These right-dominant ARVC subjects (often plakophilin-2 mutation carriers) commonly have focal LV disease involving the lateral LV wall with only mild or moderate LV dysfunction [111]. Large-scale studies from collaborative international registries are necessary to further unravel genotype-phenotype associations in ARVC.

## Update on ARVC management

ARVC management is directed towards symptom reduction, delay of disease progression, and prevention of SCD. Because of a lack of randomized trials comparing ARVC treatment options, management recommendations in ARVC are largely based on clinical judgments and results from retrospective registry-based studies. Mainstay therapies consist of conservative measures (exercise restriction), beta-blocking and antiarrhythmic agents, implantable cardioverter-defibrillator (ICD) implantation, and radiofrequency ablation of ventricular arrhythmias.

Evidence for a potential role of exercise in ARVC expression and disease progression is accumulating. Many ARVC patients are highly athletic and those who participate in competitive sports have a 5-fold increased risk of arrhythmic death compared to non-athletes [112]. Recently, James led a study on the role of exercise in ARVC development, showing that endurance exercise and frequent athletics increases the risk of arrhythmias and heart failure in ARVC mutation carriers [113]. This important piece of evidence highlights the importance of exercise restriction in ARVC patients and those at risk of developing disease.

Once the diagnosis of ARVC is established in a patient, the most important decision is whether to implant an ICD for prevention of SCD. It is now standard of care for ARVC subjects with prior sustained ventricular arrhythmia to undergo placement of an ICD [114],[115]. Studies have shown that these patients have a high incidence rate of appropriate ICD discharges of up to 70% during a mean follow-up of 3–5 years [115],[116]. Unfortunately, guidelines for ICD implantation among subjects without prior ventricular arrhythmia are less unambiguous. Recent reports suggest an important role for CMR in risk stratification of these patients [32],[117],[118]. In their study, Deac *et al.* showed that an abnormal CMR was an independent predictor of arrhythmic events [118]. Also, it was recently shown that the revised CMR TFC have high negative predictive value for arrhythmic occurrence in ARVC [32].

Arrhythmia control in ARVC is often achieved by pharmacologic treatment. Beta-blockers and class III antiarrhythmic drugs (sotalol, amiodarone) have been shown to be successful in reducing arrhythmia burden and likelihood of ICD discharge [119],[120]. In addition, radiofrequency ablation for ventricular arrhythmia in ARVC has gained enormous popularity over the last years. Although the results of endocardial ablation have been moderate [121], good arrhythmia control (but not complete cure) has been obtained using epicardial ablation [52],[122]–[124]. This is understandable, given the primary (sub)epicardial location of the abnormal substrate in ARVC. CMR with LGE may be useful in planning of these procedures, by providing information on the presence and distribution of ventricular scar [125].

## Future directions

CMR evaluation in ARVC is a moving target. New CMR sequences such as high-resolution T1 mapping are promising tools to detect early, subtle changes in the RV. In addition, quantification of RV regional wall motion abnormalities and evaluation of inter- and intraventricular dyssynchrony may provide novel tools for early detection of ARVC. The genetic era allowed for ARVC genetic testing using comprehensive cardiomyopathy panels and whole exome sequencing, which are likely to significantly impact our knowledge of the genetic basis of ARVC and the overlap with other cardiomyopathies. Furthermore, genotype-phenotype correlation studies may guide our quest for genetic and environmental modifiers in this disease. Lastly, basic research in *in vitro* and animal models may have an important impact on our knowledge of ARVC pathophysiology. Results from these studies may open the path to modification of the abnormal substrate in ARVC, allowing for definite prevention of clinical disease manifestation and/or progression.

## Conclusion

ARVC is a rare but important cause of SCD in the young and in athletes. The disease is inherited as an autosomal dominant trait with incomplete penetrance and variable expressivity. Because of the inherent risk of potentially lethal arrhythmias, correct diagnosis and early detection of ARVC are essential. This is critically important, because with the advent of genetic testing, the population of at-risk individuals is rapidly increasing. Clinical ARVC diagnosis is facilitated by a complex set of diagnostic criteria which were first described in 1994 and updated in 2010 to increase sensitivity for early disease. As the non-invasive “gold standard” for RV evaluation, CMR plays an important role in clinical ARVC workup. Recent studies have shown that RV involvement in ARVC often manifests as regional wall motion abnormality or global ventricular dysfunction, whereas LV involvement is often observed as LGE and/or fatty infiltration without concomitant wall motion abnormalities. ARVC preferentially affects the basal RV and lateral LV, while sparing the RV apex. Once diagnosis of ARVC is established, the most important management decision is whether to implant an ICD for prevention of SCD. Future studies are necessary to further unravel the pathophysiologic attributes of disease and provide insights into genotype-phenotype correlations in ARVC.

## Abbreviations

ARVC: Arrhythmogenic right ventricular cardiomyopathy

CMR: Cardiovascular magnetic resonance

DCM: Dilated cardiomyopathy

ECG: Electrocardiogram

FGRE: Fast gradient echo

ICD: Implantable cardioverter-defibrillator

LGE: Late Gadolinium enhancement

LV: Left ventricle

MESA: Multi-ethnic study of atherosclerosis

PPV: Positive predictive value

RV: Right ventricle

SCD: Sudden cardiac death

SSFP: Steady state free precession

TFC: Task Force Criteria

## Competing interests

The authors declare that they have no competing interests.

## Authors’ contributions

AT, HT, and DB conceived the study, performed the background research and review, and drafted the manuscript. All authors read and approved the final manuscript.
